# Developing machine learning algorithms for dynamic estimation of progression during active surveillance for prostate cancer

**DOI:** 10.1038/s41746-022-00659-w

**Published:** 2022-08-06

**Authors:** Changhee Lee, Alexander Light, Evgeny S. Saveliev, Mihaela van der Schaar, Vincent J. Gnanapragasam

**Affiliations:** 1 Department of Artificial Intelligence, Chung-Ang University, Seoul, South Korea; 2 Division of Urology, Department of Surgery, University of Cambridge, Cambridge, UK; 3 Department of Applied Mathematics and Theoretical Physics, University of Cambridge, Cambridge, UK; 4 Department of Electrical and Computer Engineering, University of California, Los Angeles, CA USA; 5 The Alan Turing Institute, London, UK; 6 Department of Urology, Cambridge University Hospitals NHS Foundation Trust, Cambridge, UK; 7 Cambridge Urology Translational Research and Clinical Trials Office, Cambridge Biomedical Campus, Cambridge, UK

**Keywords:** Prostate cancer, Risk factors

## Abstract

Active Surveillance (AS) for prostate cancer is a management option that continually monitors early disease and considers intervention if progression occurs. A robust method to incorporate “live” updates of progression risk during follow-up has hitherto been lacking. To address this, we developed a deep learning-based individualised longitudinal survival model using Dynamic-DeepHit-Lite (DDHL) that learns data-driven distribution of time-to-event outcomes. Further refining outputs, we used a reinforcement learning approach (Actor-Critic) for temporal predictive clustering (AC-TPC) to discover groups with similar time-to-event outcomes to support clinical utility. We applied these methods to data from 585 men on AS with longitudinal and comprehensive follow-up (median 4.4 years). Time-dependent C-indices and Brier scores were calculated and compared to Cox regression and landmarking methods. Both Cox and DDHL models including only baseline variables showed comparable C-indices but the DDHL model performance improved with additional follow-up data. With 3 years of data collection and 3 years follow-up the DDHL model had a C-index of 0.79 (±0.11) compared to 0.70 (±0.15) for landmarking Cox and 0.67 (±0.09) for baseline Cox only. Model calibration was good across all models tested. The AC-TPC method further discovered 4 distinct outcome-related temporal clusters with distinct progression trajectories. Those in the lowest risk cluster had negligible progression risk while those in the highest cluster had a 50% risk of progression by 5 years. In summary, we report a novel machine learning approach to inform personalised follow-up during active surveillance which improves predictive power with increasing data input over time.

## Introduction

Prostate cancer is the most common male malignancy in the Western world and its incidence is rising^1^. The main management conundrum arises from the sheer diversity of the disease and different clinical trajectories it can take (https://www.nice.org.uk/guidance/NG131). Balancing over-treatment and under-treatment with competing mortality are day-to-day clinical discussions across the globe, particularly given its apparent low-mortality but high prevalence (https://www.cancerresearchuk.org/about-cancer/prostate-cancer). The notion that many prostate cancers do not need immediate treatment has given rise to active surveillance (AS) becoming an increasingly mainstream management option for many men diagnosed with favourable prognosis disease^2^. It is estimated that up to 1 in 5 men with newly diagnosed prostate cancer are now managed at least initially with AS (https://www.npca.org.uk/content/uploads/2021/01/NPCA-Annual-Report-2020_-Infographic-140121.pdf/ and 2).

Modern AS has become increasingly sophisticated with the use of tri-modal monitoring to detect for changes in disease characteristics during follow-up using a combination of regular Prostate Specific Antigen (PSA) tests, multi-parametric Magnetic Resonance Imaging (MRI) as well as repeat prostate biopsies^3,4^. Risk stratification models have also allowed more streamlined scheduling of AS and tailored follow-up^5,6^. Most models, however, have been designed using only baseline clinic-pathological features and few include continuous data collection variables.

To date, a robust method to incorporate dynamic updates of future risk with continuous data from different monitoring methods (biochemistry, imaging and biopsy) during follow-up has been lacking. Machine learning applications allow independent pattern recognition without pre-programming or specifying variable interactions. As such it does not rely on traditional statistical models or constraints. We hypothesised that machine learning could derive a model incorporating diverse new observations during AS to continuously modify and update an individual’s risk of progression while on AS. Here we report the application of novel machine learning approaches to (i) allow “real-time” risk predictions that can be dynamically updated and (ii) derive temporal clusters of patients with similar future time-to-event outcomes based on their history and new updated observations.

## Results

### Cohort characteristics

**Fig. 1:**
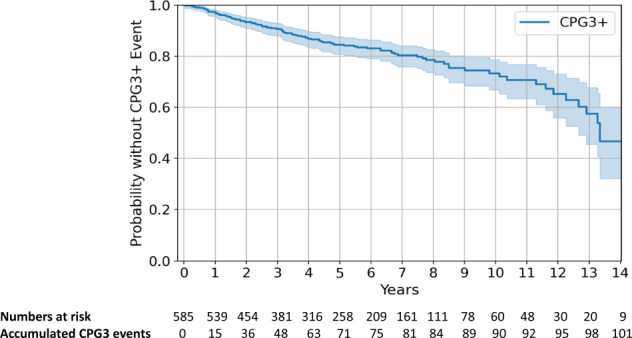
Kaplan Meier plot and confidence intervals of the cohort. The number and time to event to Cambridge Prognostic Group 3, censored or still on Active Surveillance over the period of follow-up and since the date of diagnosis.

The final cohort included 585 men with a mean age of 65.7 years, the cohort were predominantly Caucasian reflecting our patient demographics. Table 1A shows the baseline characteristics of the cohort. The majority were classified at diagnosis as CPG1 (low-risk, 68.0%). Overall median follow-up were 1601 days (4.4 years). At 3-, 5- and 10-years follow-up data was available for 65.1%, 44.1% and 10.3% of the cohort, respectively. Table 1B shows the data acquired during follow-up. PSA was the most frequent data-point available followed by serial MRI and re-biopsy (Table 1B). 17 men died of other causes during AS with no prostate cancer deaths or men progressing to metastatic disease in this series. 48 men (8.2%) went on to treatment before they reached the CPG3 + endpoint. Overall 101 (17.2%) progressed to the CPG3 + endpoint. Mean and median time to CPG3 + where this occurred was 1553 and 1150 days (4.3 and 3.2 years) respectively. Figure 1 shows the Kaplan Meier curve of events over time. Of the 101 who reached CPG3+, 21 progressed to radiological T3a, 39 had a re-biopsy upgrade to ≥Grade Group 3 and 41 had progressive PSA rises. Pathological progression (radiological + /− biopsy) therefore occurred in 50 men (8.5%) during the study period. Overall, 71.6% of men were still on AS at the end of our follow-up period.

**Table 1:** Summary of cohort and data variables collected.

Feature (static)		Percentage (%) and/or range
A		
Age at entry		65.7 years (±7.4)
Ethnicity	White	95.2%
	Others	4.8%
Family History	Yes	13.5%
	No	86.5%
Risk assignment at entry into Active Surveillance	Grade Group	Group 1: 79.9% Group 2: 20.1%
	Cambridge Prognostic Group	CPG1: 68.0% CPG2: 31%
MRI Stage at entry	Stage	T1: 26.8 % T2: 72.9 %
LIKERT Scoreat entry*	1	43.4%
	2	6.2%
	3	15.7%
	4	19.8%
	5	14.9%

Feature (time-varying)		Percentage (%) and/or range
B		
Serial PSA (ng/ml)	Mean number of observations	13.0 (±8.0)
	Mean interval between observations (days)	150.5 (range 84–182)
	Mean difference from baseline	6.44 (range ± 3.97)
Serial MRI	Mean number of observations	2.7 (±1.5)
	Mean interval between observations (days)	650.7 (range 378–662)
	MRI serial prostate volume measurements	56.88 (range ± 29.9)
	PSAd serial measurements	0.14 (range ± 0.08)
	PRECISE Scoring	1: 0.6% 2: 8.3% 3: 79.8% 4: 11.3%
Re-biopsy	Mean number of observations	1.8 (±0.8)
	Mean interval between observations (days)	766.2 (range 157–1085)
	Core Total	18.17 (±7.13)
	Core Positive	2.61 (±2.80)

A: Diagnostic static baseline covariates at Active Surveillance entry used in model development (*n* = 585 men). *Includes 69 missing values, mode imputed to 1. B: Temporal follow-up covariate data points acquired during active surveillance follow-up in model development (*n* = 585). All re-biopsies used image guided sampling of any targets on MRI as well as systematic biopsies.

### *Dynamic-DeepHit-Lite* (*DDHL*) model development and validation

**Fig. 2:**
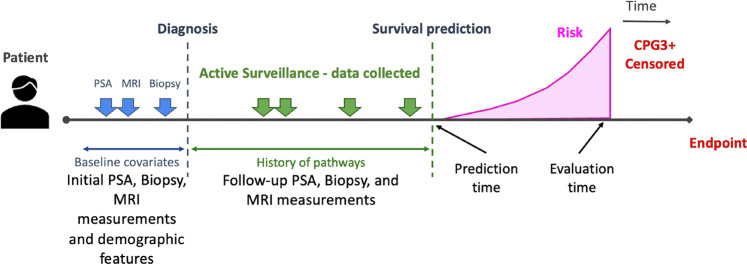
Illustration of the Dynamic-DeepHit-Lite (DDHL) survival analysis data collection method and its use in prediction modelling. CPG 3 (Cambridge Prognostic Group 3) event as the endpoint.

Figure 2 illustrates the schematic of the DDHL prediction modelling, with both baseline and follow-up covariate data collections. We first developed the *DDHL* model on a training set for time-to-event analysis and then applied it on the testing set (Table 2). Both Cox and *DDHL* models showed comparable performance with only baseline variables considered but improvement in performance of the *DDHL* model with the addition of incremental follow-up data. With 3 years of data collection and 3 years follow-up the *DDHL* model had a C-index of 0.79 (±0.11) compared to 0.70 (±0.15) for landmarking Cox and 0.67 (±0.09) for baseline Cox only. At 5 years follow-up the C-indices were 0.82 (±0.08), 0.75 (±0.08) and 0.73 (±0.09) respectively. Stated in terms of better predictions, the number of patients where the *DDHL* model predictions correctly discriminate the observed risks compared to landmarking Cox: with 3 years of data collection was 58 and 61, respectively for 3 years and 5 years follow-up (Supplementary Table 1). Model calibration was good across all models tested particularly at the 3-year follow-up horizon but not unexpectedly poorer at follow-up at 5 years (Supplementary Table 2).

**Table 2:** Model discrimination performance for prediction of progression to Cambridge Prognostic Group 3 (CPG3) event.

Method	Prediction Time	Evaluation Time	
		3 years	5 years
Cox (Standard)	From baseline	0.796 ± 0.03	0.786 ± 0.04
	+1 yr F/up data	0.760 ± 0.04	0.704 ± 0.06
	+2 yr F/up data	0.728 ± 0.10	0.701 ± 0.07
	+3 yr F/up data	0.673 ± 0.09	0.728 ± 0.09
Landmarking Cox	From baseline	0.796 ± 0.03	0.786 ± 0.04
	+1 yr F/up data	0.765 ± 0.03	0.717 ± 0.05
	+2 yr F/up data	0.764 ± 0.04	0.745 ± 0.05
	+3 yr F/up data	0.701 ± 0.15	0.745 ± 0.08
Dynamic-DeepHit-Lite	From baseline	0.778 ± 0.05	0.789 ± 0.06
	+1 yr F/up data	0.795 ± 0.05	0.740 ± 0.07
	+2 yr F/up data	0.780 ± 0.08	0.754 ± 0.09
	+3 yr F/up data	0.794 ± 0.11	0.816 ± 0.08

Time-dependent concordance indices are used and compared to standard Cox model using baseline variables only, landmarking and the Dynamic-DeepHit-Lite (DDHL) method. Prediction time refers to the period over which data was collected: at baseline and +1 to 3 years after starting active surveillance. Evaluation time is the follow-up period over which events were predicted. The results shown are averaged over 5 random training/testing splits.

### Identification of outcome-related temporal clusters

**Fig. 3:**
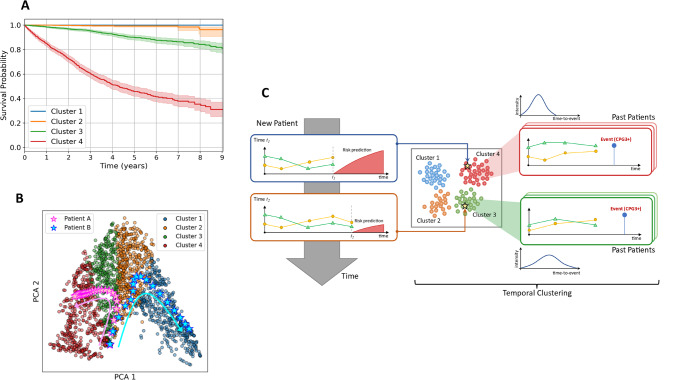
Description of the risk prediction and temporal clustering outputs and trajectories generated. **A** Kaplan Meier plot of the survival distributions and confidence intervals for the 4 temporal phenotypic clusters identified from the DDHL model showing the different rates of progression to Cambridge Prognostic Group 3 (CPG3) event over time. **B** Same temporal clusters represented in the space of the two principal components (PCA) of the latent embeddings (each point corresponds to a sampled patient). Trajectories of two illustrative example patients A and B also shown: A deteriorates as more observations are collected, moving from Cluster 3 to 4, while B improves, moving from Cluster 2 to 1. **C** A schematic illustrating the risk prediction and temporal clustering aspects of the DDHL model. (Right-hand side) Hypothetical Clusters 3 and 4 are illustrated to show the sets of past patients assigned to them in training. Cluster 4 is presented to contain higher risk patients; hence the histogram indicates an overall earlier time-to-event among these patients. Cluster 3 is presented to contain relatively lower risk patients, hence the histogram peaks at a later time-to-event. (Left-hand side) A hypothetical patient’s progression: at time t1 the patient has a comparatively steep risk prediction curve (red slope), and the model assigns him to Cluster 4; as more observations are made at time t2, the risk profile is lowered, patient is now in Cluster 3.

To further interpret the potential of the *DDHL* model, the AC-TPC method was used to discover clusters with similar time-to-event predictions. This identified that men on AS could be assigned to 4 distinct temporal clusters with different trajectories to CPG3 + progression (Fig. 3A). Those in the lowest risk cluster had small risks of progression (5% or less) even up to 8 years from starting AS (Fig. 3A). In contrast those in the highest cluster had a > 50% risk of progression by 5 years. Men can transit from one cluster to another based on new observations acquired during AS (illustrated for two examples from the cohort in Fig. 3B). Both the temporal outcome-oriented clustering, as well as the risk predictions aspects of the DDHL model are schematically illustrated in more detail in Fig. 3C. Supplementary Fig. 4 illustrates the state transition probability for a given new reading either back into the same cluster or to a cluster higher or lower. In Supplementary Fig. 5, an example is shown whereby for a given set of PSA and PSAd a patient may be at a lower or higher cluster based on the underlying Grade Group and Stage. If the PSA and PSAd progressively increases, then the patient may move to the next highest risk cluster. Alternatively, if an MRI or re-biopsy shows an upgrade in histology or upstage in disease staging, then this may also result in a cluster change for a given PSA and PSAd. The order of most contributing variable on the status of temporal cluster was Grade followed by PSA and Stage (Supplementary information Section F).

### Comparison with other AS prediction models

To benchmark performance, we compared our model and clusters to a recently reported risk stratification model based on a dynamic risk prediction model from the Canary PASS group (Supplementary Table 3)^7^. This model was developed using landmarking Cox with time to progression to ≥Grade Group 2 as the endpoint and with outputs given as percentiles of risk. For a fair head-to-head comparison, we re-constructed the Canary PASS model using our cohort and input features used to build the DDHL model and with CPG 3+ outcome as the endpoint. Our 4-cluster model provided higher discriminative power over two versions of risk stratification using the Canary PASS model (3-risk groups; lowest and highest 10th percentiles and an intermediate group; 4-risk group using 25th, 50th and 75th percentiles). This was especially apparent over longer-time horizons (Supplementary Table 3). With 3 years of data collection and 3 years follow-up the discovered clusters had a time dependent C-index of 0.92 compared to 0.62 and 0.79 with the Canary PASS 3 and 4 tier-risk models respectively; at 5 years follow-up the C-indices were 0.86 versus 0.63 and 0.78, respectively.

### Clinical utility of the demonstrator

**Fig. 4:**
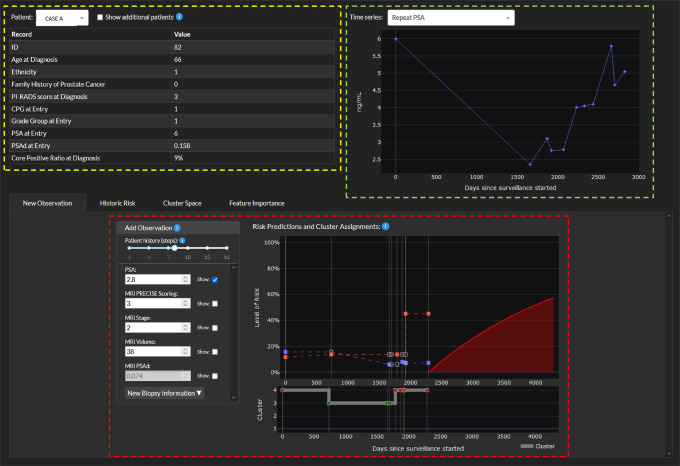
Representative image from a demonstration interface of how the DDHL and AC-TPC algorithms would work in practice. (available at https://demo-dynamic-tte.herokuapp.com). Yellow box - data display on the patient’s baseline demographics. Green box - history of sequential readings, in this display PSA readings over time since starting AS. Red box - when a new data point is entered, it generates a predicted risk (red slope, in this case this is flat) from that point onwards of developing a CPG3 event until altered by a new reading.

To demonstrate practical application of the algorithm, we developed a webtool https://demo-dynamic-tte.herokuapp.com to illustrate its potential utility in the clinic (Fig. 4). Two cases with complete histories are used for illustration. *Case A* represents a man with favourable features at AS entry for whom an event (CPG3 + ) did not occur (Fig. 4). The longer he remains stable throughout AS (*Historic risk tab*) his cluster assignment drops by a tier (Supplementary Fig. 6). The *Cluster space tab* illustrates this stable trajectory over time and down-ward movement through the clusters (Supplementary Fig. 7). Any new observations during follow-up will consider this long stable history as well as new readings to predict the risk of future progression which in this case would remain low (*New observation tab*, Supplementary Fig. 8). Based on this stability, this individual’s follow-up intervals could be reduced and less intensive. We can also model how this trajectory might have been different without this long stable history (Supplementary Fig. 9). As an example, if a new MRI had shown a change in the tumour (PRECISE 4) and a repeat biopsy showed an upgrade to Grade Group 2, his predicted risk of progression to CPG3 + increases to 6% by 3 years and 20% by 10 years (Supplementary Fig. 9). The effect of such changes during AS on outcome is further illustrated in a case where an event has occurred. *Case B* is an individual with higher risk features at the start of AS (CPG2 at entry and high PSA density). He progressed to CPG3 + on day 3092 (Supplementary Fig. 10). The *Historic risk* tab shows how at each clinic visit his risk was estimated and as repeat observations did not reduce his risk, he remained in cluster 4 throughout and eventually progressed to an event (Supplementary Fig. 11). In the *Cluster space tab*, it can be seen that he never moved away from the highest risk cluster throughout his AS. In contrast to the previous case, this individual warrant closer follow-up to ensure the progression was detected promptly.

## Discussion

To date, methods for predicting future progression in active surveillance have largely been estimated using baseline characteristics over a fixed time horizon. Here, we show that continuously collected longitudinal follow-up data can be used to inform and modify predicted trajectories of disease behaviour. In the area of AS for early prostate cancer, being able to use new real-time data as it is collected may allow tailoring of follow-up intervals, highlight a possible more aggressive trajectory, or conversely reassure about disease stability. This is particularly important in a disease which can be unpredictable and that often co-exists with other competing mortality risks in an ageing male demographic^8^.

There is a growing number of studies on machine learning methods to inform progression risks in cancer and other disease^9–11^. There are however relatively fewer studies which have looked at machine learning in predicting future cancer or progression in a surveillance scenario^12,13^. A key limitation is usually a lack of standardisation in how and what data is collected and which endpoints are relevant. Prostate cancer represents a unique oncological disease in that it is well established that non-intervention is a safe option for many early cancers and its trajectory can be monitored. This has driven the growth of structured AS programmes (incorporating serial and multi-dimensional measurement) as an important mainline management^14–17^. Thus, modern AS offers a unique opportunity to track the natural history of untreated cancer through continuously updated data collection.

AS progression calculators which include follow-up data have been developed based on standard statistical modelling^5,6^. These have mainly been used to predict an eventual histological biopsy upgrading but so far with only moderate performance and have only been developed in men with very low risk disease at diagnosis (i.e., Grade Group 1). In this study, we have observed C-indexes which surpassed these models and improved with more data collection. A novel aspect of our model is the use of a composite clinical endpoint (based on the UK National Institute of Health and Care Excellence (NICE) Cambridge Prognostic Group model). Ours is also the first model to include baseline and follow-up MRI using the relatively new PRECISE scoring system for which data and cohorts are accumulating^15,16^. Crucially using ML approaches also means we can include future new variables into the algorithms rapidly as they emerge from research findings. Beyond our own study area, the methods illustrated here may also improve other prediction models in diverse disease settings, e.g., cancer development in primary care populations, post-treatment follow-up or even in appropriate timing of treatment escalation. While landmarking models and joint models are widely used for dynamic survival analysis, both models have practical limitations: Landmarking models are “partially conditional” as the model is trained and make predictions only based on the latest observations available at the landmarking times rather than incorporating the entire history. Joint models may suffer from the model misspecification (e.g., the assumption on the underlying longitudinal process and time-to-event process) and the optimisation of joint models produces several computational challenges. Deep learning models can fully utilize the entire longitudinal trajectories using RNN specifically designed for classification or regression. However, to the best of our knowledge, there are no other deep learning methods suitable for dynamic survival analysis that can provide valid risk functions which must be a non-decreasing function of time given the longitudinal observations. Another aspect of our model is that we derived clusters in addition to individual level predictions which in our view is more clinically usable to guide management. By doing so, we considered that clinicians can more easily leverage temporal clustering as an actionable tool to (i) recognize similar past patients (for whom a pathway with an endpoint was already collected) for forecasting future events which may cause a patient to transit between clusters, and (ii) to better design follow-up strategies that are tailored to specific clusters. We recognise that using clusters as opposed to individual predictions may result in a loss in performance but we found only a small impact when comparing clusters to overall model performance (Supplementary Table 3).

Our study does have limitations. It is based on a relatively small and single centre cohort. Our median follow-up is less than 5 years though this is a clinically useful time-frame in AS terms^4,17^. We note that some variables, for example serial MRI did have a significant amount of missing data and its value in the model and whether omitting it would have made a difference is unclear. Comparison with other models was also challenging as there are none which include MRI imaging, and few which utilise dynamic information collected. Others also include variables which we do not use. For instance, the Canary PASS model incorporates body mass index, prior medication and a bespoke PSA kinetic value which is not routinely collected in AS protocols^7^. Very few have reported using ML methods and so far, have only consider baseline variables^18^. Our AS practice may also not reflect practice in other units. For example, we are more permissive in allowing more men with intermediate-risk (CPG2) disease at diagnosis into AS (38% of our cohort). However, this is becoming increasingly accepted and thus reflects more contemporary practice^17,19^. It is also acknowledged that model performance improves when predictions are across a wider risk range^20^. We also acknowledge that our AS endpoint, CPG3 + , is not a widely accepted one with most groups using any biopsy upgrading as the key metric to define progression^5–7^. However, this endpoint, unlike others, has a clear guideline recommendation based rational in terms of a benefit to switching from treatment versus continuing a conservative management approach (https://www.nice.org.uk/guidance/NG131). Progression to treatment as an endpoint for example is known to vary significantly between centres both within healthcare systems and globally as it is not standardised and subject to clinical and patient’s selection bias^21,22^. Our resultant algorithm based on the CPG3 + endpoint does therefore need robust external validation including large enough cohorts for robust statistical comparisons with other prevailing methods before any future clinical implementation or adoption. Inclusion of lifestyle factors and comorbidity (e.g., obesity, smoking) that may hasten progression could also be incorporated into the model in future work and in this regard machine learning approaches lend itself well to iterative inclusion of new variables. The results should therefore be considered hypothesis generating and an exemplar of the potential utility of machine learning algorithms and approaches to refine real-time risk prediction. Our model does, however, already appear to outperform other existing AS prediction models although this will need formal head-to-head comparison and more exploration of the most appropriate number of stratification tiers/clusters.

In summary, we present here a report of the first machine learning application of real-time temporal clustering of continuous data to inform prostate cancer active surveillance follow-up based on longitudinal observations. We demonstrate that the algorithm outperforms standard statistical tools and improves its predictive power over time. We further illustrate how it can be developed into a clinical tool that can be used in consultations and planning management in real time. Future work will explore this method and approach in more detail as a potential strategy to aid management of men on active surveillance for prostate cancer.

## Methods

### Cohort structure

The study used deidentified data from men enroled into a structured AS programme and prospectively maintained database^14,15^. The method and protocol for AS management in our centre has been previously reported in detail^14,15^. In brief, all men were diagnosed with Cambridge Prognostic Group (CPG) 1 or 2 disease (equating to low risk and favourable-intermediate risk, respectively) based on the UK National Institute for Health and Care Excellence (NICE) prognostic grouping system (https://www.nice.org.uk/guidance/NG131) (shown in Supplementary Fig. 1A). Men were diagnosed by image (MRI) guided biopsy systematic and targeted biopsy at diagnosis or, if MRI performed after diagnosis, a repeat targeted biopsy was performed within 3 months^14^. Patients underwent multi-parametric prostate MRI on a 3-T Discovery MR750 HDx or a 1.5-T MR450 scanner (GE Healthcare). Dynamic contrast-enhanced imaging was performed at baseline, but not in follow-up imaging. Prostate volume was calculated by MRI estimated volumetric measurements using the ellipsoid formula. All men on AS had 3-monthly PSA testing and annual repeat MRI with reporting done at initial diagnosis (using the 5-point Likert scoring system) and then subsequently monitored using the Prostate Cancer Radiological Estimation of Change in Sequential Evaluation (PRECISE) criteria^15^. Protocol re-biopsies were recommended at 1 and 3 years unless otherwise indicated by a change in PSA (3 consecutive rises) and/or MRI change^14^. We excluded data if there was insufficient baseline information or with less than 1-year follow-up. Supplementary Fig. S1B shows our data selection process. The study is registered with Institutional Review Board approval (Cambridge University Hospitals NHS Foundation Trust, Cambridge, UK; registration number: 3592 and 3059/PRN 9059).

### Variables and endpoints for model development

We considered the following variables for model development (i) At diagnosis: Family history, Prostate Specific Antigen (PSA), prostate volume and PSA density (PSAd) i.e., PSA divided by volume, histological Grade Group (GG), MRI stage and Likert score, biopsy core positivity (positive biopsy cores/total cores taken). PSA, PSAd, prostate volume and core positivity were modelled as continuous variables. MRI stage, Likert score, GG, and family history as categorical factors. Missing data were considered missing at random and imputed using mode (categorical variables) and mean (continuous variables) standard imputation methods (Table S4). (ii) In follow-up: PSA, PSAd, MRI PRECISE score, repeat biopsy information (GG and biopsy core positivity). Missing data were imputed based on the last observation carried forward method. Any measurements still missing were handled similar to baseline data. The pre-specified endpoint for this study was progression to ≥CPG3 disease which we have previously reported as a clinically meaningful exit criterion and consistent with NICE recommendations^10^. Progression to CPG3 + is possible through a rise in PSA, change in histological Grade Group (e.g., an increase from Grade Group 2 to Grade Group 3) or change in stage of tumour (e.g., T2 to T3) as shown in Supplementary Fig. S1A. Meeting this endpoint was, therefore, possible through 4 scenarios: (i) upgrading to any ≥GG3 disease on repeat biopsy; (ii) upgrade to GG2 disease if the PSA ≥ 10 ng/mL; (iii) progression to ≥T3 disease; (iv) or a PSA increase to ≥ 20 ng/mL. Men who did not progress were censored at the date of treatment, death, or latest investigation whichever occurred first. Time-to-event or censoring was derived from the date of diagnosis or the date of reaching to last contact: either reaching ≥CPG3 (denoted as CPG3 + ) or last follow-up.

### Model description

Dynamic-DeepHit-Lite is a deep learning-based individualised longitudinal time-to-event model was derived using *Dynamic-DeepHit* (https://github.com/chl8856/Dynamic-DeepHit). DDH learns, utilizing a recurrent neural network (RNN), the distribution of time-to-event outcomes to assess the risk of disease progression using longitudinally-linked clinical features that are systematically collected^23^. In this study, we considered clinical data collection to be (i) at diagnosis (static observations) and ii) in follow-up (longitudinal observations) concatenated into an ordered sequence according to their associated timestamps and linked to the event of reaching CPG3 + (Supplementary information Section A). Thus, the time-to-event outcome indicates the time at which a CPG3 + event occurs, or the time at which a patient is censored (Supplementary Fig. S2 and Supplementary Information Section B). To apply Dynamic-DeepHit to our relatively small dataset, the network was regularized by assuming that the underlying time-to-event process followed the Weibull distribution^24^. We modified the output layer such that the conditional intensity functions of the time-to-event process were estimated as non-linear functions of clinical pathways, which lead to a significant reduction of the number of trainable parameters. This modified approach was termed *Dynamic-DeepHit-Lite* (*DDHL*) whose network architecture is illustrated in Supplementary Fig. 2. During testing, we took the clinical pathway of a new patient as an input into *DDHL*, then utilized the output to compute the risk of having an event occurring at or before a given time elapsed since the last observation (described in Supplementary Information Section C Equation 4). For model evaluation, we report all the results averaged over 5 random 80/20 splits (80% of the study population (*n* = 468) for training the model and hold-out 20% (*n* = 117) for testing the model). Time-dependent C-indices and time-dependent Brier scores were adapted to the longitudinal observations as previously described and calculated for assessing discrimination performance and calibration performance, respectively^25^. Further details on the *DDHL* method are given in the Supplementary Methods. Model predictions were compared with standard baseline Cox and landmarking Cox methods in time-to-event analysis. To further analyse what *DDHL* learned, we utilized a temporal outcome-orientated clustering method; Actor-Critic approach for temporal predictive clustering (AC-TPC)^26^. AC-TPC discovers outcome-oriented clusters by grouping clinical pathways that share similar time-to-event outcomes despite seemingly heterogeneous progression patterns. The number of clusters is determined in a data-driven way without pre-specification. In this study, we modified AC-TPC such that it treated the trained *DDHL* algorithm as a black-box function and utilized the inputs and outputs (i.e., time-to-event predictions) to partition patients’ clinical pathways into temporal clusters (Supplementary Fig. 3). This transformed the raw information into interpretable clusters that could anticipate future behaviour using recognized similar past patient patterns either, to transit between clusters or to remain within a cluster Further details on the AC-TPC method are given in Supplementary Information Section D and E. To determine the order of contributing variable on the status of temporal cluster, we used a partial dependence plot [R1] by changing the value of each variable while fixing the values of other variables to see how the assigned temporal cluster changes with further details in Supplementary Methods Section F. To assess the performance of our model we compared it to a recently reported model from the Canary Prostate Active Surveillance Study (PASS) group which derived a percentile risk calculator^7^. The details of this comparison is provided in Supplementary Information Section G.

### Model application clinical demonstrator

The demonstrator web app was built using Plotly Dash (available at: https://github.com/plotly/dash), as it provides a flexible API for visualisation of data-focused Python projects. The app interfaces with the underlying model directly by loading it from a checkpoint and by loading cached historic predictions as needed. The design goal of the interface was to show the user an overview of a patient, as well as the various aspects of our model. The app covers: (i) risk predictions and cluster assignments spanning the patient’s history, (ii) a visualisation of a patient’s trajectory through the clusters plotted in the space of first two principal components of the latent embeddings, and (iii) provides an interactive inference tool where predictions can be made using new observations entered by the user. The demonstrator can be accessed here: https://demo-dynamic-tte.herokuapp.com.

### Reporting summary

Further information on research design is available in the Nature Research Reporting Summary linked to this article.

## Supplementary information

Supplemental information

Reporting Summary

## Data Availability

Access to de-identified data can be requested through application to the corresponding author and is subject to approval from the institutional regulatory body and approval of data sharing agreements.

## Code availability

The source code for the analysis is available at: https://github.com/vanderschaarlab/ml-as-prostate-cancer. All authors had access to the raw data and VG and AL verified the data. There was no funder organisation involved in data access, curation, or verification.
