# Supplementary material for: Developing machine learning algorithms for dynamic estimation of progression during active surveillance for prostate cancer
Source: NPJ Digit Med. 2022 Aug 6;5:110. doi: 10.1038/s41746-022-00659-w (PMC9357044; doi:10.1038/s41746-022-00659-w)
Supplement: Supplementary file 1 — Supplemental information [file 41746_2022_659_MOESM1_ESM.pdf]

Supplementary methods

Method Details

Section A. Modelling Clinical Pathways

For each patient, we define $\left((\mathbf{x}_j, t_j)_{j=1}^{J}, \Delta, \tau\right)$ to be a variable-length clinical pathway, which comprises J longitudinal observations where $x_j \in \mathbb{R}^d$ denotes the d-dimensional observed features including both static and time-varying covariates, $t_j \in \mathbb{R}_{\geq 0}$ is the timing at the j-th observation, $\Delta \in \{0,1\}$ indicates whether the patient has experienced the event ($\Delta = 1$) or the patient is right-censored ($\Delta = 0$), and $\tau \in \mathbb{R}_{\geq 0}$ is the time at which the event or censoring occurred, respectively. For ease of notation, we often denote the history of observations up to time t_i as $\mathcal{X}(t_i) = (\mathbf{x}_j, t_j)_{j=1}^{i}$. Note that irregular time intervals between observations can be generally described by the actual timestamps t_j.

Define $T \in \mathbb{R}_{\geq 0}$ be a random variable for the time-to-event and $C \in \mathbb{R}_{\geq 0}$ be a random variable for the time-to-censoring. We assume that T, C are drawn from a conditional distribution that depends on the history of a patient's longitudinal observations, respectively, and we only observe the event or the censoring that occurs first, i.e., $\Delta = \mathbb{I}_{\{T \leq C\}}$ and $\tau = \min(T, C)$. Then, the *conditional hazard function* $h(s|\mathcal{X}(t_i))$ [6], which represents the instantaneous risk of the outcome event occurring given the history $\mathcal{X}(t_i)$, can be defined as:

$$h(s|\mathcal{X}(t_i)) = \lim_{ds \to 0} \frac{P(s \leq T \leq s + ds|\mathcal{X}(t_i), T \geq s)}{ds} \tag{1}$$

where s denotes the time elapsed since the latest observation time t_i. Then, we can express the probability of the time-to-event outcome (Δ, τ) given the history $\mathcal{X}(t_i)$ as the following: $P(T = \tau - t_i|\mathcal{X}(t_i)) = h(\tau - t_i|\mathcal{X}(t_i))S(\tau - t_i|\mathcal{X}(t_i))$ if event m occurred (i.e., $\Delta = 1$) and $P(T > \tau - t_i|\mathcal{X}(t_i)) = S(\tau - t_i|\mathcal{X}(t_i))$ if right-censored (i.e., $\Delta = 0$). Here, $S(s|\cdot) = \exp\left(\int_0^s h(u|\cdot)du\right)$ is the survival function which captures the probability of a patient's event-free survival up to s.

Now, we assume that the conditional hazard functions follow the *Weibull* distribution [2], which is one of the most common parametric forms to analyze time-to-event processes. That is, given the history $\mathcal{X}(t_i)$, (1) can be simplified as:

$$h(s|\mathcal{X}(t_i)) = p\lambda(\mathcal{X}(t_i))\left(\lambda(\mathcal{X}(t_i))s\right)^{p-1} \tag{2}$$

where $\lambda(\mathcal{X}(t_i)) > 0$ is the *conditional intensity function* given $\mathcal{X}(t_i)$ and $p > 0$ is the shape parameter.[1] Then, given a clinical pathway $\mathcal{X}(t_i)$, we can derive the risk of having an event occur at or before time s elapsed since the last observation time t_i as

[1] The Weibull distribution is a generalization of the exponential distributions. For instance, when $p = 1$, it reduces to the standard exponential distribution and has constant hazard function over time, while the hazard function is i ncreasing and decreasing over time when $p > 1$ and $p < 1$, respectively.

$$R(s|\mathcal{X}(t_i)) = 1 - \exp(-(\lambda(\mathcal{X}(t_i))s)^p) \quad (3)$$

The risk, $R(s|\mathcal{X}(t_i))$, denotes the probability of an event occurring at or before time s given the input pathway up to timestamp i. It is worth highlighting that whenever a new observation is collected Dynamic-DeepHit-Lite re-issues the risk predictions that start from 0 due to the fact that this patient is alive at the time at which the new observation is collected.

The log-likelihood of a patient's clinical pathway can be derived as $\sum_{j=1}^{J} \log P\left(T = \tau - t_j \middle| \mathcal{X}(t_j)\right)$ where the conditional probability of an outcome event and the timing (Δ, τ) given the history $\mathcal{X}(t_j)$ in the log-likelihood of the outcome sequence can be derived as follows:

$$\begin{aligned}
\log P(T = \tau - t_i|\mathcal{X}(t_i)) &= \mathbb{I}_{\{\Delta=1\}} \cdot \log\left(h(\tau - t_i|\mathcal{X}(t_i))S(\tau - t_i|\mathcal{X}(t_i))\right) + \mathbb{I}_{\{\Delta=0\}} \cdot \log S(\tau - t_i|\mathcal{X}(t_i)) \\
&= \mathbb{I}_{\{\Delta=1\}} \cdot \log h(\tau - t_i|\mathcal{X}(t_i)) + \log S(\tau - t_i|\mathcal{X}(t_i)) \\
&= \mathbb{I}_{\{\Delta=1\}} \cdot \log\left(p\lambda(\mathcal{X}(t_i))(\lambda(\mathcal{X}(t_i))(\tau - t_i))^{p-1}\right) - \lambda(\mathcal{X}(t_i))^p(\tau - t_i)^p
\end{aligned}$$

Hence, the problem of accurately estimating the log-likelihood of a time-to-event outcome boils down to accurately estimating the conditional intensity functions $\lambda(\cdot)$ as a function of the clinical pathway.

Section B. Modeling TTE Processes via NNs

We use an RNN to model the underlying dynamics of the time-to-event outcomes given clinical pathways. The key idea here is to determine the conditional intensity functions in (2) from the latent representations (i.e., the hidden states) of the RNN. This allows learning of complex dependencies of the conditional hazard functions on the history of observations. The network comprises an *encoder* that captures the underlying dynamics given a pathway and a *predictor* that estimates the conditional intensity functions based on the output of the encoder. The biggest distinction of Dynamic-DeepHit-Lite from the original work in [1] comes from modeling the time-to-event process as the Weibull distribution.

The encoder, $f^{\theta}: \prod_{j=1}^{i}(\mathbb{R}^d \times \mathbb{R}_{>0}) \rightarrow \mathcal{Z}$, is an RNN (parameterized by θ) that takes a sequence of tuples $\mathcal{X}(t_i)$ – i.e., the pathway that contains available observations and the timing up to the i-th time step – as inputs and maps the input sequence to latent representations $\mathbf{z}_i$, $f^{\theta}(\mathcal{X}(t_i)) \in \mathcal{Z}$ at each time step i. Note that, owing to the RNN structure, our model can flexibly handle the longitudinal data with each subject having different numbers of observations that are measured with irregular time intervals. Utilizing the Gated Recurrent Unit (GRU) [7], $\mathbf{z}_i$ can be derived as follows:

$$\mathbf{h}_i = \sigma(W_h\mathbf{z}_{i-1} + U_h[\mathbf{x}_i\ t_i] + \mathbf{b}_h),$$

$$\mathbf{r}_i = \sigma(W_r\mathbf{z}_{i-1} + U_r[\mathbf{x}_i\ t_i] + \mathbf{b}_r),$$

$$\tilde{\mathbf{z}}_i = tanh(W_z(\mathbf{r}_i \circ \mathbf{z}_{i-1}) + U_z[\mathbf{x}_i\ t_i] + \mathbf{b}_z),$$

$$\mathbf{z}_i = (1 - \mathbf{h}_j) \circ \mathbf{z}_{i-1} + \mathbf{h}_j, \circ \tilde{\mathbf{z}}_i,$$

where W, U, and $\mathbf{b}$ are weight matrices and vector which parameterize the encoder, $\circ$ is element-wise multiplication, $\sigma(\cdot)$ is the sigmoid function, and $tanh(\cdot)$ is the tangent function.

The predictor, $f^{\phi}: \prod_{j=1}^{i} \mathcal{Z} \rightarrow \mathbb{R}_{>0}$, is a fully-connected network (parameterized by ϕ) that estimates the conditional intensity functions in (2) given the latent representation of the input sequence at each time step i, that is, $\lambda(\mathcal{X}(t_i)) \stackrel{\text{def}}{=} f^{\phi}(\mathbf{z}_i) = f^{\phi}\left(f^{\theta}(\mathcal{X}(t_i))\right)$.

Section C. Dynamic Time-to-Event Outcome Predictions

Our primary goal is to issue the risk of a patient of having the event of interest given the patient's clinical pathway. To do so, we utilize the output of Dynamic-DeepHit-Lite to re-write the risk function in (3) as:

$$R(s|\mathcal{X}(t_i)) = 1 - \exp\left(-\left(f^{\phi}\left(f^{\theta}(\mathcal{X}(t_i))\right) s\right)^{p}\right) \tag{4}$$

where s is the time elapsed since the latest observation time t_i.

Section D. Outcome-Oriented Temporal Clustering

Given the trained Dynamic-DeepHit-Lite, we now focus on discovering temporal clusters that characterize the underlying disease progression in terms of the predictions on the time-to-event outcomes made by the trained Dynamic-DeepHit-Lite based on patients' clinical pathways. To this goal, we modify AC-TPC such that it treats the trained Dynamic-DeepHit-Lite as a black-box function and utilizes the inputs and outputs (i.e., time-to-event predictions) of Dynamic-DeepHit-Lite to partition patients' clinical pathways into temporal clusters that share similar time-to-event predictions. More specifically, we formalize temporal clustering defined in [5] as learning discrete representations that best characterize the underlying time-to-event process learned by Dynamic-DeepHit-Lite through the pathways. The key insight here is that learning embeddings (i.e., a finite number of latent representations available for discrete representation learning) and the mappings from pathways to these embeddings can be viewed as learning the centroids of each cluster (i.e., the representative representations of each cluster) and the assignments of the pathways to these clusters, respectively.

Let $s_i \in \{1, \dots, K\}$ be the cluster assignment at time step i and $\mathcal{E} = \{\mathbf{e}(1), \dots, \mathbf{e}(K)\}$ where $\mathbf{e}(k) \in \mathcal{Z}$ be the *embedding dictionary*. Then, we define $\bar{\mathbf{z}}_i \stackrel{\text{def}}{=} \mathbf{e}(s_i) \in \mathcal{Z}$ to be the *embedding*, a discrete representation of clinical pathways in the latent space. At each time step i, the discrete representation can be obtained as follows: first, we find an *encoding* $\hat{\mathbf{z}}_i = g^{\theta}(\mathcal{X}(t_i))$ (i.e., a continuous representation in the latent space) of an input pathway $\mathcal{X}(t_i)$ as an output of the AC-TPC encoder. Then, based on the encoding $\hat{\mathbf{z}}_i$, the cluster assignment s_i is drawn from a categorical distribution defined by the AC-TPC selector output, i.e., $s_i \sim Cat(\boldsymbol{\pi}_i)$ where $\boldsymbol{\pi}_{\mathbf{i}} = [\pi_i(1), \dots, \pi_i(K)] \stackrel{\text{def}}{=} g^{\theta}(\bar{\mathbf{z}}_i)$. Once the cluster assignment s_i is chosen, we allocate the latent encoding $\hat{\mathbf{z}}_i$ to an embedding $\bar{\mathbf{z}}_i$ as described above. Finally, we can estimate the conditional intensity function as $\bar{\lambda}(\mathcal{X}(t_i)) \stackrel{\text{def}}{=} g^{\theta}(\bar{\mathbf{z}}_i)$.

Given the conditional intensity functions given the cluster assignment and those estimated by the trained Dynamic-DeepHit-Lite, we can compute the JS-divergence between the two time-to-event processes as the following[2]:

$$JS(\lambda||\bar{\lambda}) = \frac{1}{2}\left(\frac{\lambda(\mathcal{X}(t_i))}{\bar{\lambda}(\mathcal{X}(t_i))}\right)^p + \frac{1}{2}\left(\frac{\bar{\lambda}(\mathcal{X}(t_i))}{\bar{\lambda}(\mathcal{X}(t_i))}\right)^p - 1 \quad (5)$$

Finally, we replace the loss functions in [5] with the newly defined divergence (5) and train the modified AC-TPC to discover the outcome-oriented temporal clusters based on the time-to-event predictions of the trained Dynamic-DeepHit-Lite.

Section E. Hyper-Parameters of DDHL and AC-TPC

For the network architecture of DDHL, we construct the encoder (f^{θ}) utilizing two-layer GRU with 100 nodes in each layer, and the predictor (f^{ϕ}) and the selector (f^{ψ}) utilizing three-layer fully-connected network with 100 nodes in each layer. The parameters (θ, ϕ, ψ) are initialized by Xavier initialization and optimized via Adam optimizer with learning rate 0.001 and dropout with keep probability 0.6. All the balancing coefficients are chosen based on the grid search over the possible set of values as suggested in [1] and [5] based on the validation loss (i.e., 20 percent of the training set is used when choosing the balancing coefficients).

Section F: Partial dependence plot to determine the order of contributing variables on cluster movement

A partial dependence plot was used to change the value of each variable while fixing the values of other variables to see how the assigned temporal cluster changes [8]. Since the three variables – PSA, MRI Stage, and Grade – are not in the same scale and with different categories, we plotted the average effect on the cluster status in Figure F1-F3. In these figures, the transition frequency implies the frequency of making a transition to a higher risk cluster (e.g., from Cluster 2 to Cluster 3) when positive, and that of making a transition to a lower risk cluster (e.g., from Cluster 2 to Cluster 1) when negative. As can be seen in the figure, the order of most contributing variable on the status of temporal cluster is Grade, PSA, and Stage.

[2] We use JS-divergence between the two Weibull distributions instead of using KL-divergence in the original AC-TPC [5] due to the symmetric property.

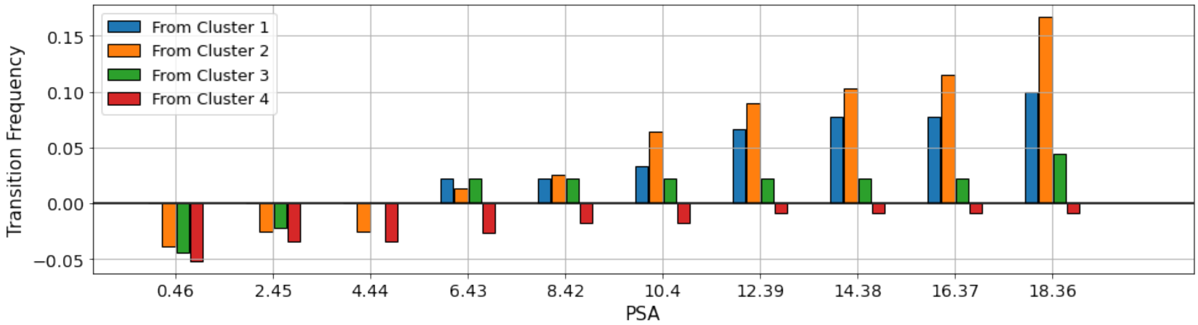

Figure F1

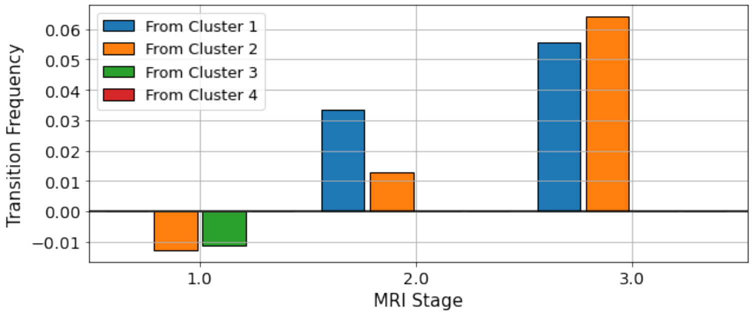

Figure F2

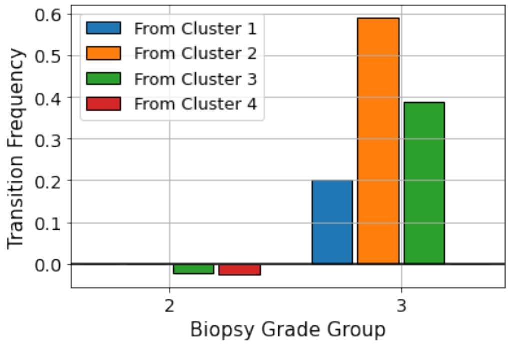

Figure F3

Section G. Benchmarks and comparison with the Canary-PASS risk stability model

We compare our model against the commonly used methods in the medical setting: Cox proportional hazards [8], [9] at baseline (using static covariates only) and landmarking Cox [10], [11] (using both static and temporal covariates up to the prediction times) in the dynamic setting. The full set of features is used, as with Dynamic-DeepHit-Lite, and the regularization parameter α is set to 1e-3. For the landmarking Cox, we set the landmarking times as $L = 0, 1, 2,$ and 3 years.

For evaluating discriminative performance, we use time-dependent concordance index for right-censored data based on inverse probability of censoring weights [13] throughout; for calibration performance evaluation, time-dependent Brier score [14] is computed.

When evaluating the discriminative performance to compare the discovered clusters and simple stratification approaches, we use the time-dependent concordance index as above, with cluster index or stratum index (higher index corresponding to higher risk group) used as risk estimates. The time-to-event models, including DDHL, LM-Cox, and static Cox, make risk predictions whose value is between 0 and 1. Thus, we use those outcomes directly to evaluate the discrimination and prediction performance. In contrast, the clustering methods, including AC-TPC and Canary-PASS, predicts to which cluster a patient belongs based on his longitudinal observations. We compared the "discriminative power" of the two clustering methods by using the average predicted risks for each cluster, as an indirect way to compare how similar the patients are within a cluster and how dissimilar the patients are across different clusters. Considering the description above, we provided the best comparison that we could to compare the two clustering methods. More specifically, when building the Canary-PASS model, we first trained a LM-Cox using the same training set; in particular, time-to-event information was also provided to build Canary-PASS model during training. To provide a fair comparison with respect to the discriminative power of the discovered groups (i.e., clusters in the proposed method and stratifications of Canary-PASS model), we wanted to match the numbers of groups. Hence the results of the Canary-PASS as a 4 strata model, which is the same number of clusters discovered by our method, were utilized.

Supplementary methods reference list

[1] C. Lee, J. Yoon, and M. van der Schaar, “Dynamic-deephit: A deep learning approach for dynamic survival analysis with competing risks based on longitudinal data,” IEEE Transactions on Biomedical Engineering, April 2019.

[2] D. W. Hosmer, S. Lemeshow, and S. May, Applied Survival Analysis: Regression Modeling of Time-to-Event Data. John Wiley & Sons, Inc, 2008.

[3] Uno, H., Cai, T., Pencina, M. J., D’Agostino, R. B., & Wei, L. J. (2011). “On the C-statistics for evaluating overall adequacy of risk prediction procedures with censored survival data”. Statistics in Medicine, 30(10), 1105–1117..

[4] U. B. Mogensen, H. Ishwaran, and T. A. Gerds, “Evaluating random forests for survival analysis using prediction error curves,” Journal of Statistical Software, vol. 50(11), 2012.

[5] C. Lee and M. van der Schaar, “Temporal phenotyping using deep predictive clustering of disease progression,” In Proceedings of the 37th International Conference on Machine Learning (ICML 2020), 2020.

[6] R. L. Prentice, J. D. Kalbfleisch, J. A. V. Peterson, N. Flournoy, V. T. Farewell, and N. E. Breslow, “The analysis of failure times in the presence of competing risks,” Biometrics, vol. 34(4), pp. 541–554, 1978.

[7] J. Chung, C. Gulcehre, K. Cho, and Y. Bengio, “Empirical evaluation of gated recurrent neural networks on sequence modeling,” arXiv preprint arXiv:1412.3555, 2014.

[8] J. H. Friedman, “Greedy function approximation: A gradient boosting machine,” Ann. Statist., vol. 29, no. 5, pp. 1189–1232, 2001

[9] C. R. David en Others, “Regression models and life tables (with discussion)”, Journal of the Royal Statistical Society, vol 34, no 2, bll 187–220, 1972.

[10] N. Breslow, “Covariance analysis of censored survival data”, Biometrics, bll 89–99, 1974.

[11] Y. Zheng en P. J. Heagerty, “Partly conditional survival models for longitudinal data”, Biometrics, vol 61, no 2, bll 379–391, 2005.

[12] H. C. Van Houwelingen, “Dynamic prediction by landmarking in event history analysis”, Scandinavian Journal of Statistics, vol 34, no 1, bll 70–85, 2007.

[13] S. Pölsterl, “scikit-survival: A Library for Time-to-Event Analysis Built on Top of scikit-learn”, Journal of Machine Learning Research, vol 21, no 212, bll 1–6, 2020.

[14] H. Uno, T. Cai, M. J. Pencina, R. B. D’Agostino, en L.-J. Wei, “On the C-statistics for evaluating overall adequacy of risk prediction procedures with censored survival data”, Statistics in medicine, vol 30, no 10, bll 1105–1117, 2011.

[15] E. Graf, C. Schmoor, W. Sauerbrei, en M. Schumacher, “Assessment and comparison of prognostic classification schemes for survival data”, Statistics in medicine, vol 18, no 17–18, bll 2529–2545, 199

Cambridge Prognostic Group	Criteria
1	Gleason score 6 (grade group 1)<br>**and**<br>prostate-specific antigen (PSA) less than 10 microgram/litre<br>**and**<br>Stages T1–T2
2	Gleason score 3 + 4 = 7 (grade group 2) **or** PSA 10 microgram/litre to 20 microgram/litre<br>**and**<br>Stages T1–T2
3	Gleason score 3 + 4 = 7 (grade group 2) **and** PSA 10 microgram/litre to 20 microgram/litre **and** Stages T1–T2<br>**or**<br>Gleason 4 + 3 = 7 (grade group 3) **and** Stages T1–T2
4	One of: Gleason score 8 (grade group 4), PSA more than 20 microgram/litre, Stage T3
5	Two or more of: Gleason score 8 (grade group 4), PSA more than 20 microgram/litre, Stage T3<br>**or**<br>Gleason score 9 to 10 (grade group 5)<br>**or**<br>Stage T4

A.

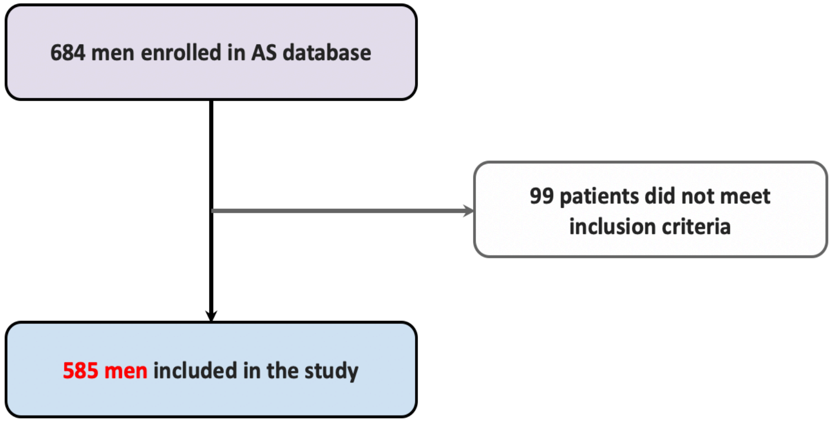

B.

Supplementary Figure 1. A. The UK National Institute for Health and Care Excellence (NICE) prognostic grouping criteria used in this study (https://www.nice.org.uk/guidance/ng131/chapter/recommendations). **B.** An illustration of data selection and assembly before analysis.

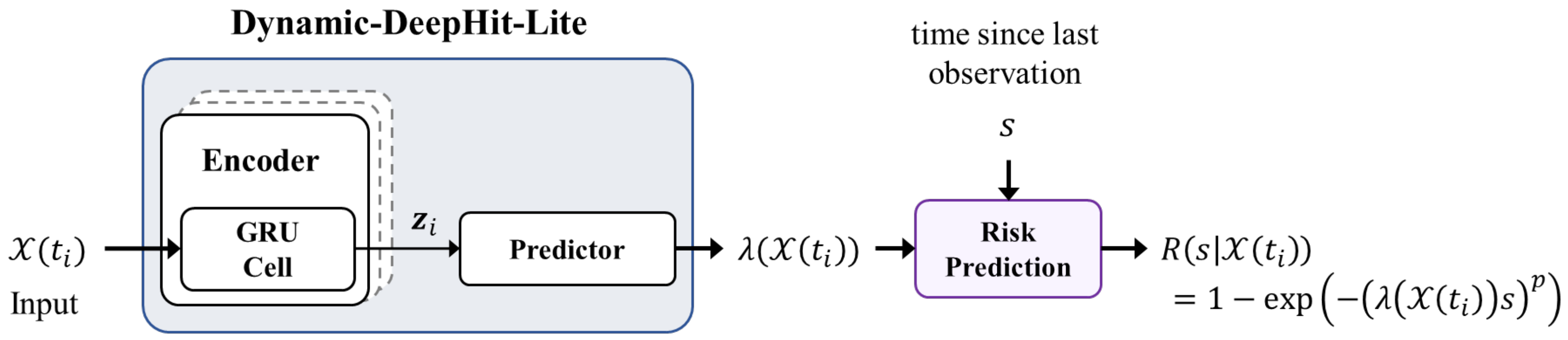

Supplementary Figure 2. An illustration of the network architecture of the Dynamic-DeepHit-Lite (DDHL) method.

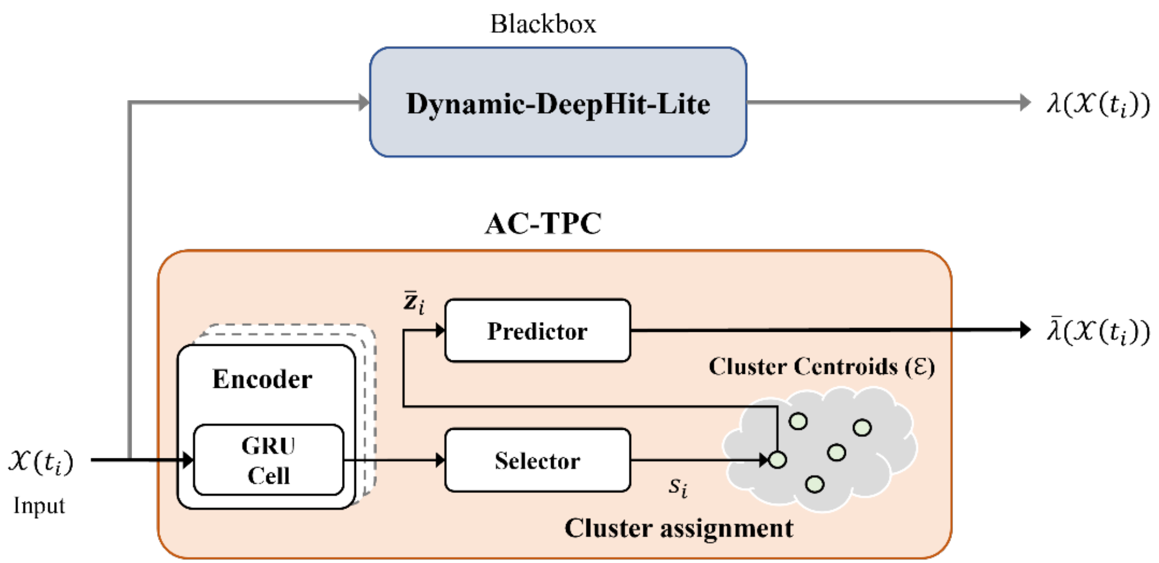

Supplementary Figure 3. An illustration of Actor-Critic approach for temporal predictive clustering (AC-TPC).

Method	Prediction Time	Evaluation Time			
		3 years		5 years	
**Dynamic-DeepHit-Lite**	From baseline	472/574	(+ 12)	875/1012	(+ 28)
	+ 1 yr F/up data	781/914	(+ 72)	903/1054	(+ 64)
	+ 2 yr F/up data	466/518	(+ 71)	524/587	(+ 71)
	+ 3 yr F/up data	444/480	(+ 58)	471/507	(+ 61)

Supplementary Table 1. Number of correctly ordered pairs / number of acceptable pairs] for prediction of progression to Cambridge Prognostic Group 3 (CPG3) for the Dynamic-DeepHit-Lite (DDHL) method. The value in red brackets is the number of correctly ordered pairs compared to the landmarking Cox model. Evaluation time is the follow-up period over which events were predicted. Results shown here are for one particular training/testing set split, as the number of acceptable pairs varies between data splits.

Method	Prediction Time	Evaluation Time	
		3 years	5 years
**Cox (Standard)**	From baseline	**0.079 ± 0.02**	**0.114 ± 0.02**
	+ 1 yr F/up data	0.091 ± 0.01	0.132 ± 0.03
	+ 2 yr F/up data	0.076 ± 0.02	0.136 ± 0.03
	+ 3 yr F/up data	0.085 ± 0.03	0.126 ± 0.04
**Landmarking Cox**	From baseline	**0.079 ± 0.02**	**0.114 ± 0.02**
	+ 1 yr F/up data	0.093 ± 0.01	**0.131 ± 0.02**
	+ 2 yr F/up data	0.076 ± 0.02	0.136 ± 0.03
	+ 3 yr F/up data	0.090 ± 0.03	0.148 ± 0.04
**Dynamic-DeepHit-Lite**	From baseline	0.084 ± 0.02	0.120 ± 0.02
	+ 1 yr F/up data	**0.089 ± 0.00**	0.134 ± 0.04
	+ 2 yr F/up data	**0.074 ± 0.02**	**0.129 ± 0.03**
	+ 3 yr F/up data	**0.077 ± 0.03**	**0.105 ± 0.03**

Supplementary Table 2 Brier scores in model calibration for prediction of progression to Cambridge Prognostic Group 3 (CPG3) event comparing standard Cox model using baseline variables only, landmarking and the Dynamic-DeepHit-Lite (DDHL) method. Prediction time refers to the period over which data was collected: at baseine and + 1 to 3 years after starting AS (standard Cox model only used data at baseline). Evaluation time is the follow-up period over which events were predicted.

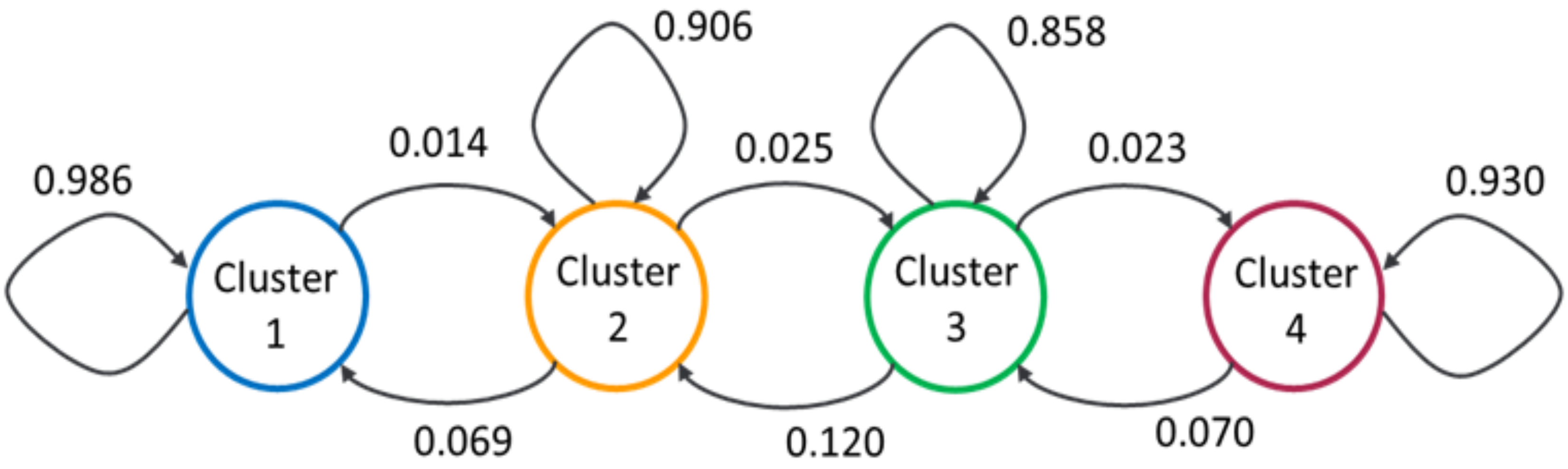

Supplementary Figure 4. Cluster transition diagram illustrating scenarios of the population-level probability of making a transition from one cluster to the other. Each number represents the average probability of transitions to the same cluster or a cluster higher or lower based on the individual disease trajectory and a new data point. For example, if a patient is currently in Cluster 3, on average, this man will make a transition to Cluster 2 with a probability of 0.120, Cluster 4 with a probability of 0.023, and will stay in Cluster 3 with a probability of 0.858. The transition probabilities (i.e., the probabilities of cluster assignments) are different for each individual patient which may also change over time within a single patient.

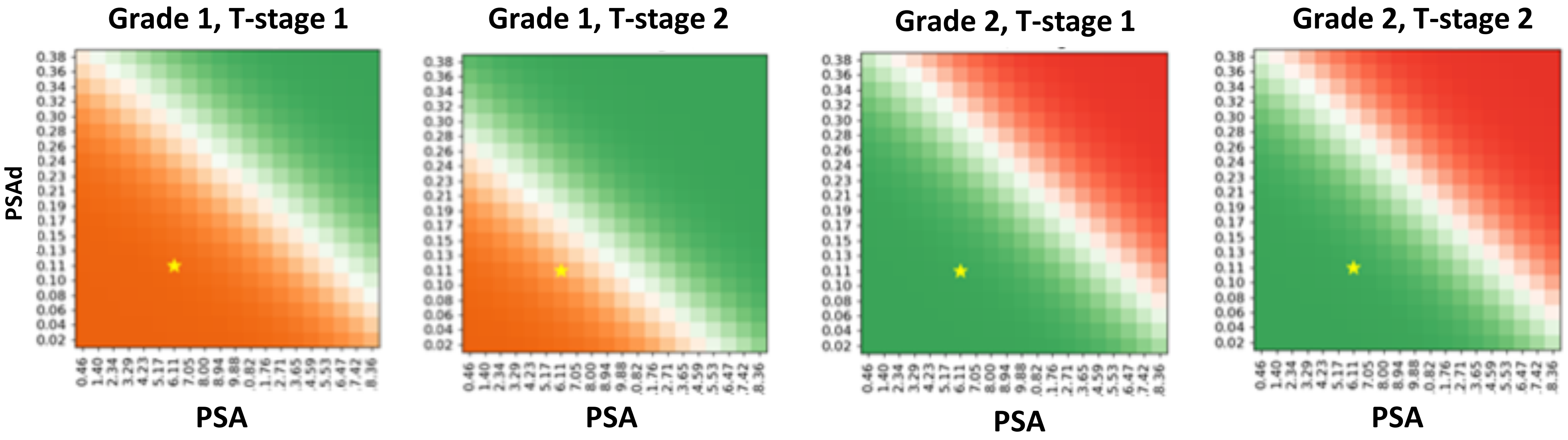

Supplementary Figure 5. Illustration of how temporal phenotypic cluster assignments may change (yellow star) based on given set of datapoints: in this example the next PSA (x-axis) and PSA density PSAd (y-axis) for patient scenarios with different baseline Grade Group and Stage is shown (Blue: Cluster 1 (lowest risk- not seen in this case), Orange: Cluster 2, Green: Cluster 3, Red: Cluster 4 (highest risk).

Method	Prediction Time	Evaluation Time	
		3 years	5 years
**Canary-PASS 3 risk strata***	From baseline	0.625	0.597
	+ 1 yr F/up data	0.624	0.594
	+ 2 yr F/up data	0.733	0.646
	+ 3 yr F/up data	0.629	0.632
**Canary-PASS 4 risk strata****	From baseline	0.697	0.727
	+ 1 yr F/up data	0.708	0.705
	+ 2 yr F/up data	0.783	0.745
	+ 3 yr F/up data	0.794	0.782
**Dynamic-DeepHit-Lite**	From baseline	**0.704**	**0.751**
	+ 1 yr F/up data	**0.765**	**0.739**
	+ 2 yr F/up data	**0.794**	**0.769**
	+ 3 yr F/up data	**0.920**	**0.867**

Supplementary Table 3. Discrimination comparison versus risk stratified deciles in the Canary-PASS calculator (16) for prediction of progression to Cambridge Prognostic Group 3 (CPG 3) event. For simplicity the Canary Pass model was tested with 2 scenarios: * a 3-risk group stratification model categorized by the lowest and highest 10th percentiles and an intermediate risk group from 10th to 90th percentile; ** a 4-risk group stratification model evenly divided using 25th, 50th, and 75th percentiles. Prediction time refers to the period over which data was collected: at baseline and + 1 to 3 years after starting AS (standard Cox model only used data at baseline). Evaluation time is the follow-up period over which events were predicted. In the DDHL model comparing the derived 4-cluster versus overall model predictions with 3 years of data collection and 3 years follow-up showed a difference in C-index of 0.920 versus 0.925 suggesting only a modest impact on model performance.

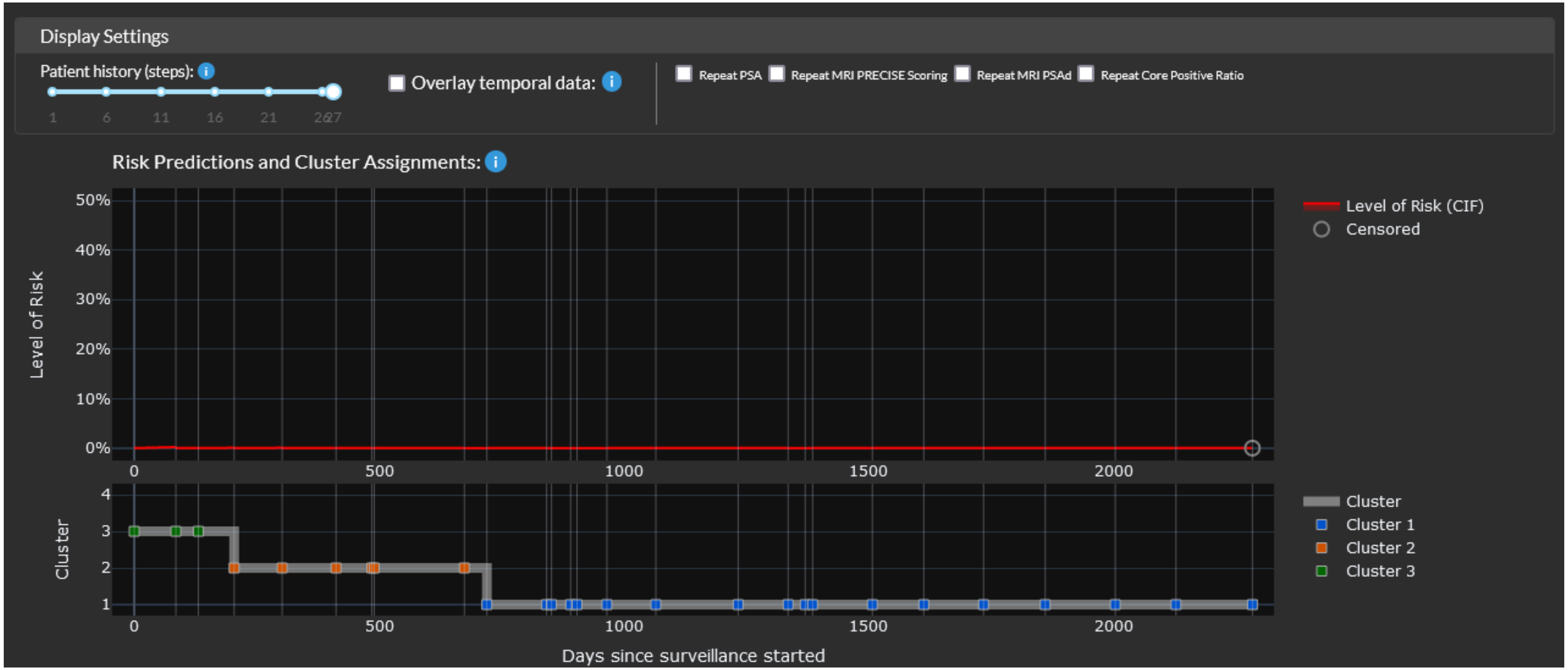

Supplementary Figure 6. Representative image from a demonstration interface of the DDHL model showing the historic view available at https://demo-dynamic-tte.herokuapp.com/. *Case A* shown here represents a man who has been on stable AS for some time with no progression or change.

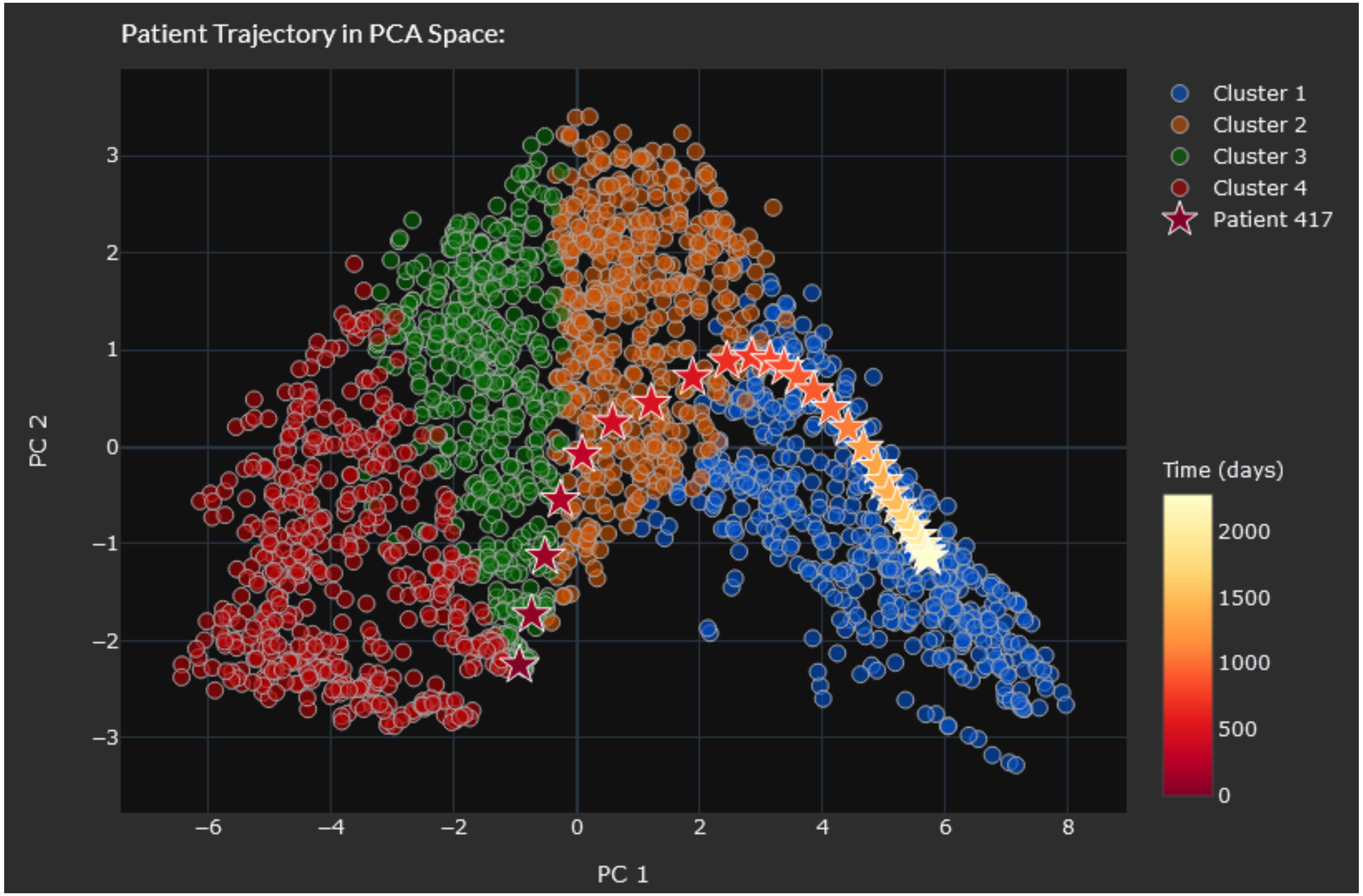

Supplementary Figure 7. Representative image from a demonstration interface for *Case A* who had a long period of stable observations and no progression to CPG3. *Cluster space tab* illustrates his trajectory over time and progressive movement through the clusters and eventually to the lowest risk cluster with a prolonged period of no change.

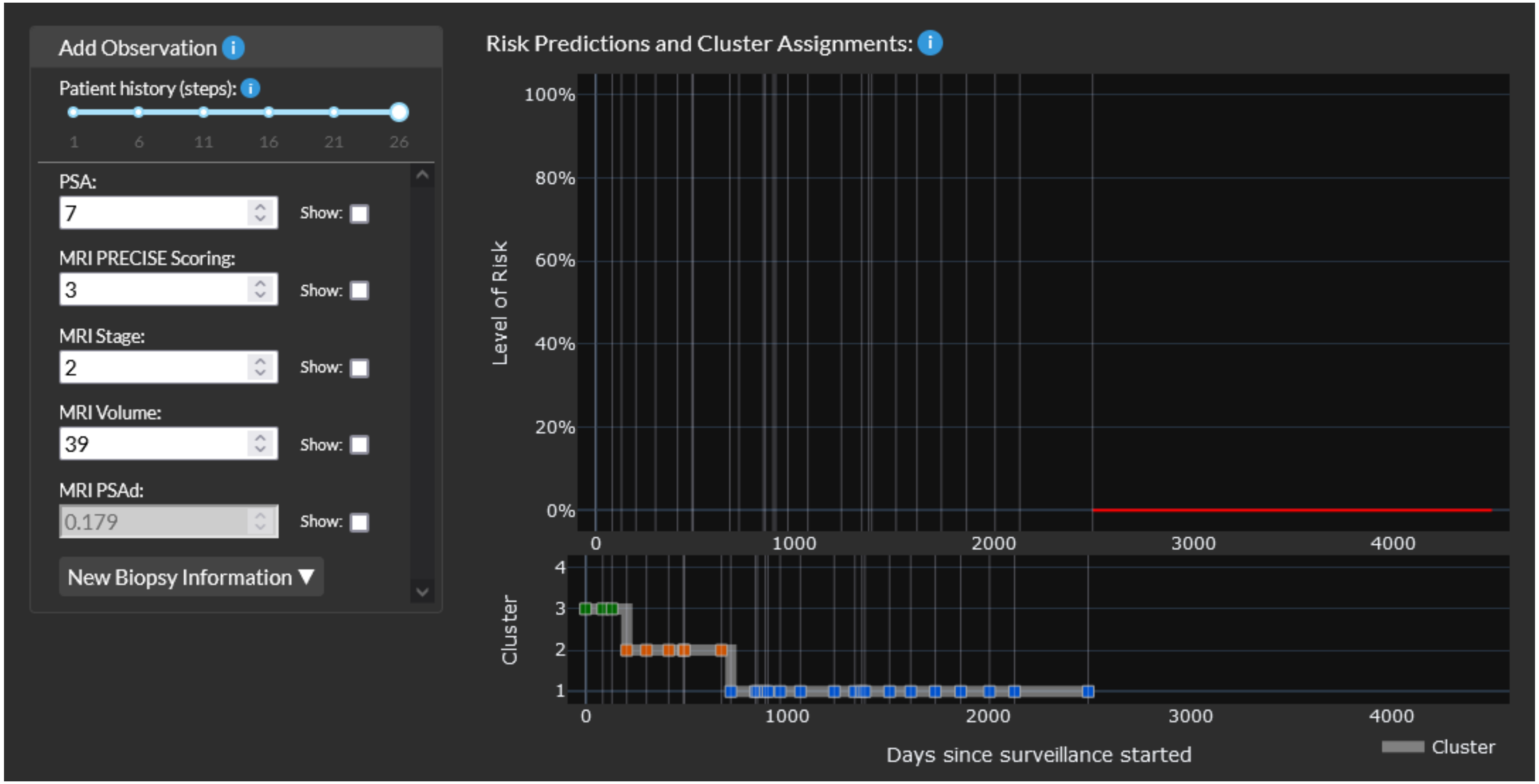

Supplementary Figure 8. *New observations tab* for *Case A*. A new observation can be added and the future risk recalculated taking into consideration the long stable history. If new observations are added with higher risk features, the overall change of progression is higher but is mitigated by the long stable period on AS.

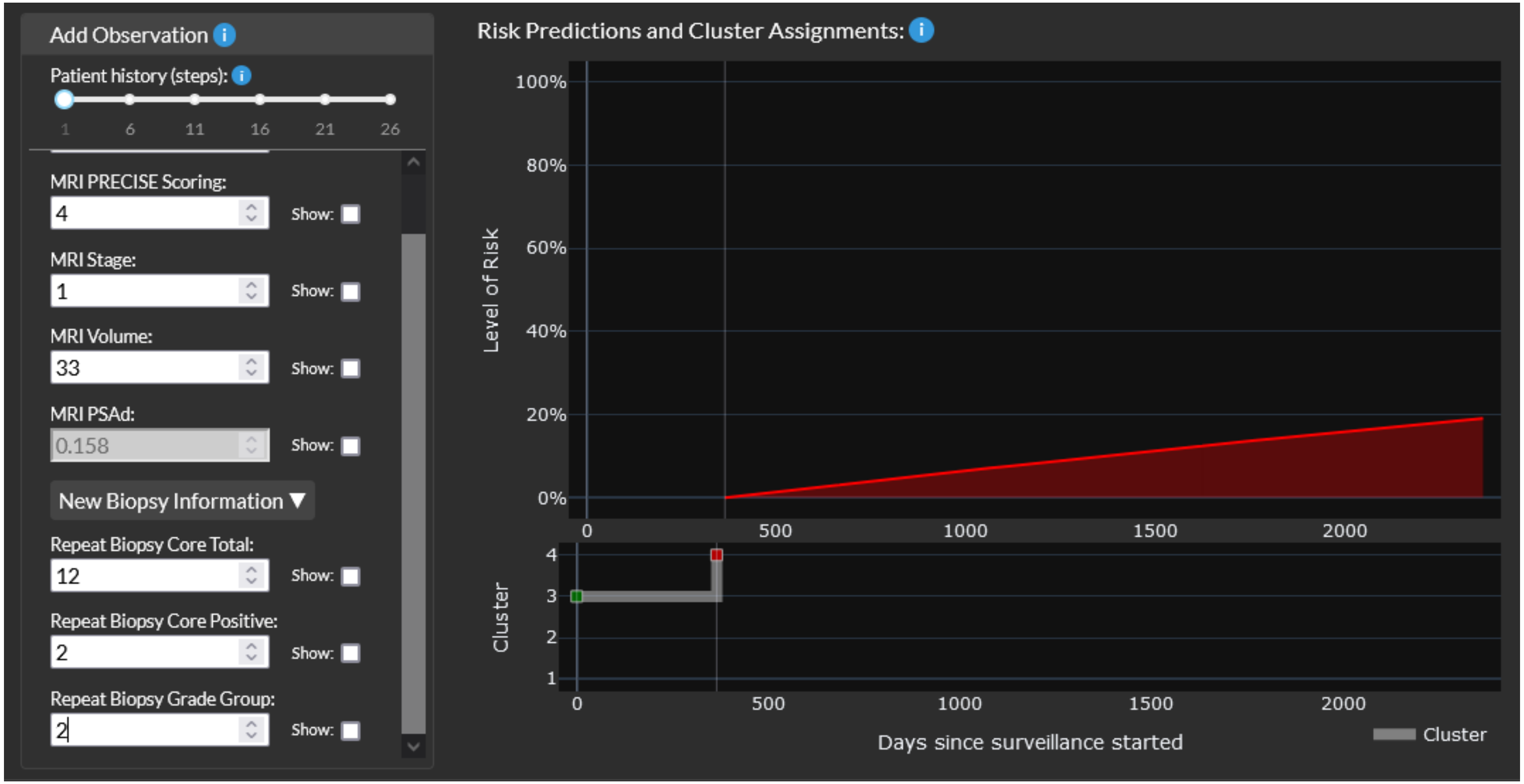

Supplementary Figure 9. Effect of changing the observations in *Case A* in the *New Observations tab* using higher risk features and without the long antecedent of stable observations on AS. It can be seen that the patient progresses to a higher cluster group and the predicted risk of a CPG3 event rises over time instead of remaining stable.

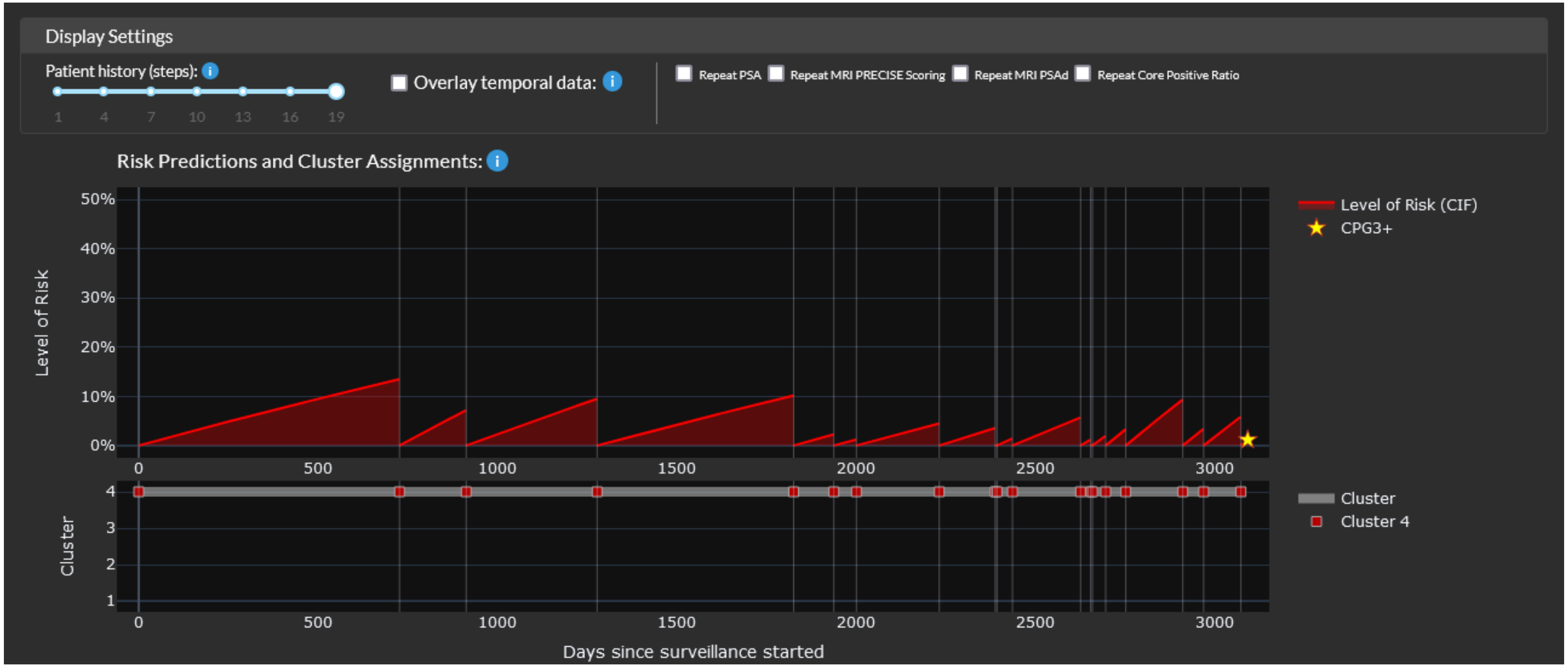

Supplementary Figure 10. Demonstration interface of the DDHL model showing the *Historic risk tab* of *Case B* - a man who has been on AS and progressed to CPG3. Over time each observation suggested a risk of progression which is reset with a new observation. Because of the ongoing risk he remains in the same (highest) risk cluster. This would prompt closer surveillance than in the previous discussed case.

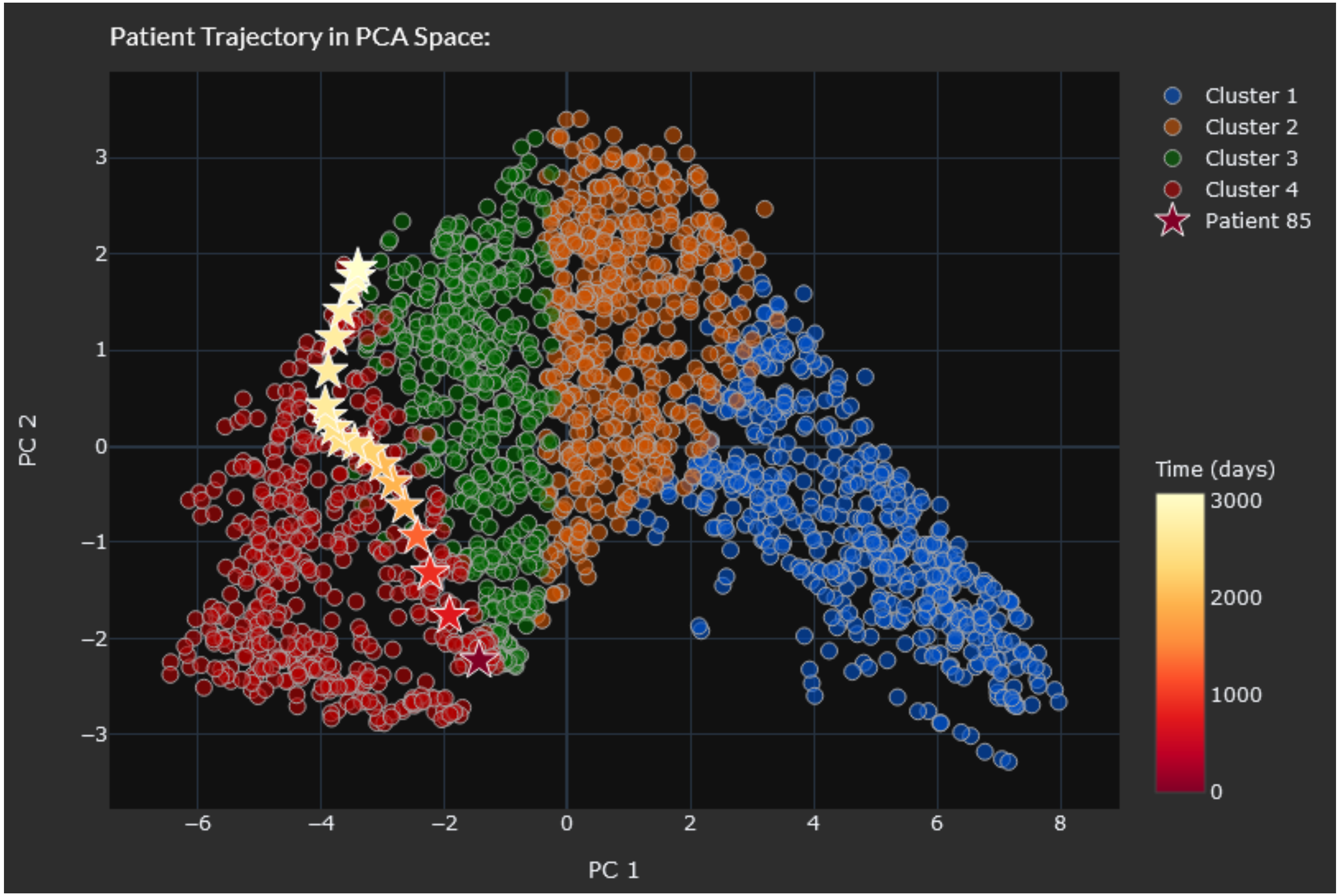

Supplementary Figure 11. Representative image from a demonstration interface for *Case B* who had a higher risk of progression at the outset for progression to CPG3. *Cluster space tab* illustrates the patient remained in the highest cluster as observations remained the same or predicted a more likely progression course.
